# Online social network size is reflected in human brain structure

**DOI:** 10.1098/rspb.2011.1959

**Published:** 2011-10-19

**Authors:** R. Kanai, B. Bahrami, R. Roylance, G. Rees

**Affiliations:** 1 UCL Institute of Cognitive Neuroscience, 17 Queen Square, London WC1N 3AR, UK; 2 Wellcome Trust Centre for Neuroimaging, University College London, 12 Queen Square, London WC1N 3BG, UK; 3 Interacting Minds Project, Institute of Anthropology, Archaeology, Linguistics, Aarhus University, Norrebrogade 44, Building 10 G, 8000 Aarhus, Denmark; 4 Centre of Functionally Integrative Neuroscience, Aarhus University Hospital, Norrebrogade 44, Building 10 G, 8000 Aarhus, Denmark; 5 Institute of Cancer, Barts and The London School of Medicine and Dentistry, Charterhouse Square, London EC1M 6BQ, UK

**Keywords:** social networks, brain structure, individual differences, personality, superior temporal sulcus, middle temporal gyrus

## Abstract

The increasing ubiquity of web-based social networking services is a striking feature of modern human society. The degree to which individuals participate in these networks varies substantially for reasons that are unclear. Here, we show a biological basis for such variability by demonstrating that quantitative variation in the number of friends an individual declares on a web-based social networking service reliably predicted grey matter density in the right superior temporal sulcus, left middle temporal gyrus and entorhinal cortex. Such regions have been previously implicated in social perception and associative memory, respectively. We further show that variability in the size of such online friendship networks was significantly correlated with the size of more intimate real-world social groups. However, the brain regions we identified were specifically associated with online social network size, whereas the grey matter density of the amygdala was correlated both with online and real-world social network sizes. Taken together, our findings demonstrate that the size of an individual's online social network is closely linked to focal brain structure implicated in social cognition.

## 1. Introduction

Web-based social network services such as *Facebook* or *MySpace* consist primarily of a representation of each user and their social links [1]. The most popular site, *Facebook*, at the time of writing has over 750 million users worldwide including almost 38 per cent of the UK adult population. These services allow individuals to articulate and make visible their friendship networks, and it is apparent that there is considerable variability in the size of such networks [2]. However, the basis for this variability and whether it reflects the size of real-world social networks remains unclear and often controversial in the absence of empirical data.

One possibility is that variability in the size of online social networks has a neural basis. For real-world human social networks, a significant amount of variability is accounted for by genetic factors [3]. The influence of these heritable factors on social networks is presumably mediated through their impact on the brain and cognition. Moreover, it has been suggested that cortical volume limits information processing capacity for the number of social relationships that an individual can monitor simultaneously [4,5]. Consistent with this, a recent study found that the size and complexity of real-world social networks correlated specifically with the volume of the left and right amygdala [6]. However, that study did not consider online social network size and the relatively small number of participants (*n* = 58) may have limited power to detect associations between social network size and other brain regions. We therefore hypothesized that the degree to which individuals participate in online social networks might be reflected in the anatomical structure of human brain regions implicated in socio-cognitive behaviours.

It is sometimes anecdotally said that ‘friends’ acquired through online social networking are of a different character or number from those acquired through real-world social networks. This raises a concern that the cognitive functions that support a large network size on *Facebook* may not necessarily correspond to those for offline, intimate social networks. Social networks for different functions (close friends, work colleagues, etc.) are organized at different scales up to the so-called ‘Dunbar number’ based on the correlation between cortical volume and group size across primate species [4,5], but these numbers are typically correlated across individuals. We therefore hypothesized that if the cognitive functions that support a large network size on *Facebook* correspond to those for offline, intimate social networks, then the number of friends on Facebook should be correlated to the number of friends an individual reports in real-world social networks.

The primary goal of the present study was to identify brain regions associated with an individual's *online* social network size. We hypothesized that brain regions mediating social cognition and memory were particularly relevant to online social network size. In the case of offline real-world social networks, perspective-taking and memory capacity abilities predict an individual's network size [7]. Thus, we hypothesized that *online* social network size might be correlated with brain regions implicated in social cognition and social behaviour such as recognizing social cues, mentalizing (i.e. theory-of-mind) and perspective-taking. A large body of evidence indicates that processing of basic social signals such as gaze and body movements of others is mediated by the posterior superior temporal sulcus (STS) [8]. The amygdala is another central structure in social cognition [9], as amygdala damage impairs the ability to recognize emotional facial expressions [10]. Indeed, an association between social network size and the amygdala has been demonstrated for real-world social networks [6]. However, whether this finding generalizes to *online* social networks is currently unknown.

In addition to superficial recognition of social signals, deeper understanding of other people's mental states via mentalizing [11,12] or the mirror circuit [13] would also be important for successful social interactions. Such high-level social cognition is associated with activity in a network of brain regions including the temporoparietal junction (TPJ), medial prefrontal cortex and precuneus [11,12,14,15]. While the precise contribution of each of those regions to social cognition is yet to be established, they are consistently activated in a broad range of tasks embedded in social contexts. Since the ability to understand other people's intention is a key to successful social interactions, the size of online social networks might be reflected in the structure of these specific social brain regions.

In addition to these brain regions linked with social cognition, memory capacity is another important constraint on the size of social network, because maintenance of a large number of social ties requires memory for relationships [16,17]. Although previous work did not find association between real-world social network size and the hippocampus [6], memory capacity may become more relevant for online social networks than real-world social network, because the number of friends declared on online social networks is much larger than that of typical real-world social networks. Of particular interest are brain regions in the medial temporal lobe (MTL), which are linked with the encoding and retrieval of face–name pairs [18,19].

To test these hypotheses, we collected structural magnetic resonance imaging (MRI) scans from a large sample of 125 healthy adult volunteers and a further replication sample of 40 volunteers. We aimed to determine whether variability in the structure of specific regions of human cortex was associated with inter-individual variability in the number of social relationships as indexed by Facebook (the *Facebook number* or FBN). To do this, we used voxel-based morphometry (VBM) to compute regional grey matter volume across the whole brain based on T1-weighted anatomical MRI scans [20]. A premise of the VBM approach is that inter-individual differences in a behavioural trait across individuals can be correlated with differences in grey matter volume of specific brain regions (see Kanai & Rees [21] for a recent review). These macroscopic measures of brain anatomy have been successfully used to identify specific brain regions that are associated with individual differences in a broad range of contexts such as perceptual performance [22,23], attention control [24], face recognition skills [25], introspective ability [26], proficiency in a second language [27], personality traits [28] and political orientation [29]. We therefore expected that this VBM approach would reveal brain regions associated with an individual's online social network size.

**Figure 1.**
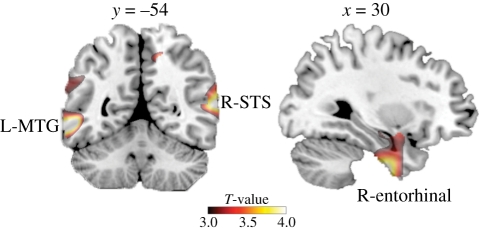
Grey matter volume correlated with quantitative measure of participation in social networks. Areas where the number of friends reported on Facebook correlated significantly with variability in grey matter density across the entire group (*n* = 125) are shown superimposed on a standard T1-weighted template brain in MNI stereotactic space. Correlated areas are shown at *T* > 3.0 for visualization purposes. See table 1 for full details of activated loci. L-MTG, left middle temporal gyrus.

## 2. Material and methods

### (a) Experiment 1

Experiment 1 determined whether inter-individual variability in the number of social relationships as indexed by Facebook (the FBN) predicted variability in brain structure.

#### (i) Magnetic resonance imaging participants

One hundred and twenty-five healthy participants (46 males; mean age 23.3 ± 4.2 (s.d.) years) recruited from the local community of University College London took part in experiment 1. After MRI scanning (see below), the number of friends for that individual in their Facebook public profile was recorded.

#### (ii) Magnetic resonance imaging data acquisition

MR images were acquired on a 1.5 T Siemens Sonata MRI scanner (Siemens Medical, Erlangen, Germany). High-resolution anatomical images were acquired using a T1-weighted three-dimensional Modified Driven Equilibrium Fourier Transform (MDEFT) sequence (TR = 12.24 ms; TE = 3.56 ms; field of view = 256 × 256 mm; voxel size = 1 × 1 × 1 mm).

#### (iii) Voxel-based morphometry

The MR images were first segmented for grey matter (GM) and white matter (WM) using the segmentation tools in SPM8 (http://www.fil.ion.ucl.ac.uk/spm). Subsequently, we performed Diffeomorphic Anatomical Registration Through Exponentiated Lie Algebra (DARTEL) in SPM8 for inter-subject registration of the GM images [30]. To ensure that the local grey matter volume was conserved after spatial transformation, the image intensity of each voxel was modulated by the Jacobian determinants of the deformation fields. The registered images were smoothed with a Gaussian kernel (full width at half maximum = 12 mm) and were then transformed to Montreal Neurological Institute (MNI) stereotactic space using affine and nonlinear spatial normalization implemented in SPM8 for multiple regression analysis.

A multiple regression analysis was performed on the smoothed grey matter images in SPM8 to determine regions in which grey matter density showed a positive correlation with the number of friends on Facebook. The gender and age of the participants and the total grey matter volume across the whole brain were included in the design matrix as covariates of no interest to model and thus regress out any effects correlated with these factors. We used *p* < 0.05 family-wise error corrected for the whole-brain volume as the criterion to detect voxels with a significant correlation with FBN.

In addition to this exploratory whole-brain analysis, which required a conservative statistical threshold to correct for multiple comparisons across the whole brain, we conducted a more sensitive region-of-interest analysis based on the previous finding that the size of bilateral amygdalae correlates with real-world social network size [6]. This previous finding served as a prior hypothesis to specifically examine correlation in the amygdala. For this purpose, we analysed the correlation within the small volumes defined by spheres (10 mm radius) centred at the MNI coordinates of amygdala as provided by Harvard–Oxford Subcortical Structural Atlas (MNI coordinates for left amygdala: *x* = −24, *y* = −2, *z* = −22; right amygdala: *x* = 26, *y* = 0, *z* = −22). By this small volume correction analysis [31], statistical results were corrected for multiple comparisons only within the small spheres. Thus, the sensitivity to detect correlation within those volumes was increased.

#### (iv) Skewness correction

The distribution of the number of friends on Facebook was highly skewed (skewness = 1.18). This could induce strong bias from a small number of participants who reported extremely large FBN. We applied the square-root transformation to the raw data to correct the strong skew. With this transformation, the violation of normality assumption owing to the skewness was adequately corrected (skewness = 0.04).

### (b) Experiment 2

The findings from experiment 1 were obtained using a conservative whole-brain correction for multiple comparisons (apart from the amygdala). To provide additional protection against the possibility of false-positive findings, in experiment 2, we sought to replicate our results in an independent sample of 40 new participants. We used the coordinates of the three locations identified in the first experiment (above) and bilateral amygdala to examine whether the results of experiment 1 were replicated in an independent dataset.

#### (i) Voxel-based morphometry replication

We collected MR images from 40 new participants who did not participate in the first VBM study (23 males; mean age 22.1 ± 3.0 (s.d.) years). Grey matter density was extracted at the three loci of interest (MNI: *x* = −57, *y* = −51, *z* = −6; *x* = 63, *y* = −54, *z* = 10; *x* = 30, *y* = −10, *z* = −42), which were identified by the initial VBM results. In addition, we also extracted the grey matter density at stereotactic coordinates corresponding to the amygdala using Harvard–Oxford Subcortical Structural Atlas (MNI coordinates for left amygdala: *x* = −24, *y* = −2, *z* = −22; right amygdala: *x* = 26, *y* = 0, *z* = −22). To be consistent with the methods used in experiment 1 (above), the factors of age, gender and the total grey matter volume were regressed out before the stage of extracting local grey matter density at these loci.

### (c) Experiment 3

To examine the relationship between the online social networks and real-world social networks, in experiment 3 we examined whether the FBN in a sample of 80 of our participants correlated with other smaller real-world social network sizes [32] revealed by a previously developed and validated Social Network Size Questionnaire [33,34].

#### (i) Social network size questionnaire

Eighty participants were recruited from the UCL student community (28 males; mean age 22.1 ± 2.9 (s.d.) years). The questionnaire was adapted from Stileman & Bates [33]. It consisted of the following nine questions.

— How many were present at your 18th or 21st birthday party?
— If you were going to have a party now, how many people would you invite?
— What is the total number of friends in your phonebook?
— Write down the names of the people to whom you would send a text message marking a celebratory event (e.g. Birthday, Christmas, new job, good exam result, etc.). How many people is that?
— Write down the names of people in your phonebook you would meet for a chat in a small group (one to three people). How many people is that?
— How many friends have you kept from school and university whom you could have a friendly conversation with now?
— How many friends do you have on ‘Facebook’?
— How many friends do you have from outside school or university?
— Write down the names of the people of whom you feel you could ask a favour and expect to have it granted. How many people is that?

These questions are loaded strongly onto a single factor [33]. We applied the square-root transformation before computing the correlations to correct for strong skewness present in the distributions.

### (d) Experiment 4

We examined how offline social network sizes measured in experiment 3 were related to the regions identified in our VBM analysis (experiment 1). To do so, we extracted the grey matter density at the loci identified in experiment 1 and bilateral amygdala from a total of 65 participants who completed the social network questionnaire in experiment 3 and were scanned either in experiment 1 or in experiment 2 (43 males; age 22.4 ± 3.3 (s.d.) years old). The method and coordinates used for grey matter extraction were identical to those used in the replication study (experiment 2).

We computed a normalized real-world social network size for each participant by averaging the *z*-scores for the question items 1, 2, 4, 5, 6, 8 and 9 after skewness correction for each item by square-root transformation (see the methods for experiment 1). These items were selected based upon their similar averages ranging between 10 and 20, which correspond to the scale of support clique [35]. The excluded question items were one about Facebook and one about phonebook, as both of these showed much larger averages and the Facebook number needed to be separated. Even when we included the question about the number of friends in their phonebook, the results were qualitatively unchanged.

In this analysis, we examined three types of relationships for each brain region of interest. First, we sought to replicate the results of experiments 1 and 2 by using the subsample of the participants. Since our sample was simply a subset of the original population, replication of the basic findings was expected. However, this was a critical step for interpretation of the analyses that included the real-world social network size as a covariate. Second, we examined whether the score of real-world social network size correlated with grey matter density of the loci that were identified in our VBM analysis (experiment 1). This analysis allowed us to determine whether the identified regions reflected only online social network size or also generalized to offline social network size. Third, we conducted a multiple regression analysis to compute partial correlations between FBN and grey matter density while regressing out the contribution of real-world network size. This analysis revealed regions that were specifically associated with online social network size. We also computed partial correlations between real-world network size and grey matter density while regressing out the factor of FBN. This analysis revealed regions that were specifically associated with real-world social network size.

## 3. Results

### (a) Experiment 1

Experiment 1 tested the hypothesis that inter-individual variability in the number of social relationships as indexed by Facebook (the FBN) predicted variability in brain structure. In accordance with our hypothesis, we found a significant positive correlation between grey matter density across individuals for three remarkably focal cortical locations (figure 1): left middle temporal gyrus (MTG; *T*_121_ = 4.16, *R* = 0.354, *p* < 0.05 family-wise error (FWE)-corrected for whole-brain volume), right posterior STS (*T*_121_ = 4.10, *R* = 0.349, *p* < 0.05 FWE-corrected for whole-brain volume) and the right entorhinal cortex (*T*_121_ = 4.09, *R* = 0.348, *p* < 0.05 FWE-corrected for whole-brain volume). Table 1 provides full details of these loci.

**Table 1.** MNI coordinates and statistical results are summarized for the peak voxels (shown in bold) and local maxima within the clusters that correlated significantly with the number of friends on Facebook (*p* < 0.05, corrected for multiple comparisons). FWE, family-wise error.

region	H	MNI coordinates (mm)			*T* values	*Z* scores	*p*_FWE-Corr_
		*x*	*y*	*z*			
MTG	**L**	**−57**	**−54**	**−6**	**4.16**	**4.02**	**<0.05**
		−63	−45	34	3.81	3.69	0.09
		−62	−60	13	3.49	3.40	0.22
STS	**R**	**63**	**−54**	**10**	**4.09**	**3.95**	**<0.05**
		66	−48	24	3.85	3.73	0.08
		66	−51	−8	3.27	3.19	0.35
entorhinal cortex	**R**	**30**	**−10**	**−42**	**4.10**	**3.96**	**<0.05**
		20	−7	−36	4.04	3.90	<0.05
		26	−4	−45	3.93	3.81	0.06

Real-world social network size, as measured by the Social Network Index [36], is correlated with the grey matter volume of the amygdala in a sample of 58 individuals [6]. In our larger sample of 125 individuals and using FBN as a measure of social network size, we did not observe any significant correlation with amygdala volume at our whole-brain-corrected threshold. However, for completeness, we re-examined our data using a more sensitive region-of-interest analysis based on the stereotactic coordinates (see §2 for full details). Using this more sensitive analysis, we found a weak but significant relationship between amygdala volume and online social network size for both right (*T*_121_ = 3.74, *R* = 0.322, *p* = 0.001 FWE-corrected for small volume) and left (*T*_121_ = 3.43, *R* = 0.298, *p* = 0.003 FWE-corrected for small volume) amygdala. It is noteworthy that the strength of the correlation we found between FBN and amygdala grey matter volume was comparable with that found previously [6].

### (b) Experiment 2

**Figure 2.**
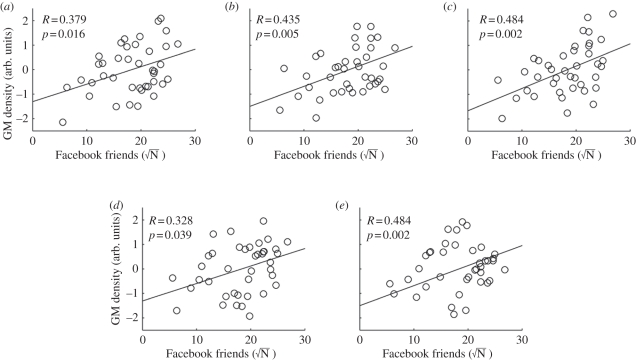
Replication of the main results with an independent dataset (*n* = 40). The relationships between the number of Facebook friends and the normalized grey matter (GM) density at the peak coordinates for (*a*) left MTG, (*b*) right STS, (*c*) right entorhinal cortex, (*d*) left amygdala and (*e*) right amygdala are plotted (see table 1 for stereotactic coordinates and figure 1 for visualization of these loci from first exploratory study). Normalized grey matter density was computed for the peak voxel coordinates after regressing out the factors of age, gender and total grey matter volume, as for the main VBM analysis (see §2 for full details). The scale of grey matter density was normalized by computing the *z*-scores across the 40 participants. In all of the three regions, significant (*p* < 0.05) correlations were observed, replicating the main VBM findings (figure 1) with an independent dataset.

Experiment 2 sought to replicate the findings of experiment 1 in a new sample of 40 individuals. In all the three cortical loci of interest showing whole-brain-corrected relationships with online social network size (i.e. from experiment 1 left MTG, right STS and right entorhinal cortex), we observed significant (*T*_38_ = 2.52, *R* = 0.379, *p* < 0.05; *T*_38_ = 2.97, *R* = 0.435, *p* < 0.01; *T*_38_ = 3.41, *R* = 0.484, *p* < 0.01, respectively) correlation between the FBN and local grey matter density in those areas (figure 2). In addition, we examined the association between online social network size and bilateral amygdala. We found significant correlation between the FBN and local grey matter density of bilateral amygdala (left amygdala: *T*_38_ = 2.14, *R* = 0.328, *p* < 0.05; right amygdala: *T*_38_ = 3.41, *R* = 0.484, *p* < 0.01). Thus, all of our findings in the first VBM analysis on 125 participants were replicated in an independent dataset.

### (c) Experiment 3

**Figure 3.**
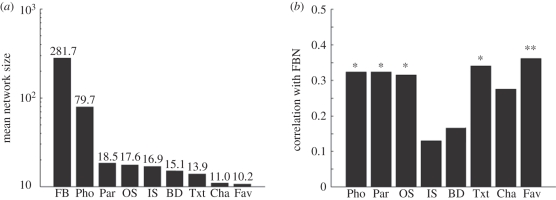
Results of the social network size questionnaire. (*a*) Geometric mean of individual's social network size is plotted for the nine item questions in the questionnaire, namely, FBN (number of friends on Facebook), Pho (number of friends in their phonebook), Par (number of friends to be invited to a party), OS (number of friends outside school), IS (number of friends from school), BD (number of friends present at birthday party), Txt (number of friends one would send a text message for a celebratory event), Cha (number of friends one would meet for a chat) and Fav (number of friends one could ask a favour). Note that the mean network size is much larger for FB. (*b*) Correlation between number of friends on Facebook and other measures. Statistical significance is denoted by asterisks (*p < 0.05 and **p < 0.01, Bonferroni-corrected for multiple comparison across the eight scores). Correlation was computed based on the square root of the declared number of friends for each question to correct for strong skew in the distribution. Influences of age and gender were regressed out by computing partial correlations.

Experiment 3 examined the relationship between FBN and the size of a variety of real-world social networks measured using standardized questionnaires. We found that across the individuals we studied, the FBN was indeed larger than the real-world network sizes for a variety of situations revealed by our questionnaire (figure 3*a*). Nevertheless, FBN exhibited significant correlation with five out of the eight measures of real-world social network sizes (even with a stringent Bonferroni correction, *p* < 0.05, *n* = 80; figure 3*b*).

### (d) Experiment 4

We examined the relationship between real-world social network size and the grey matter density at the loci identified by our VBM analysis (see experiment 1) in a subset of 65 participants who completed the social network size questionnaire (experiment 1) and MRI scan (either experiment 1 or experiment 2). Within this subsample, we first investigated the relationship between grey matter density and FBN at the loci indentified by our VBM analysis. The results replicated significant correlations between FBN and grey matter density at left MTG (*T*_63_ = 2.18, *R* = 0.264, *p* < 0.05), right STS (*T*_63_ = 2.69, *R* = 0.322, *p* < 0.01) and right entorhinal cortex (*T*_63_ = 2.43, *R* = 0.292, *p*<0.05). However, we did not find significant correlations at the amygdala loci in this particular sub-sample (left: *T*_63_ = 1.08, *R* = 0.135, *p* = 0.283; right: *T*_63_ = 1.35, *R* = 0.182, *p* = 0.168), although the direction of the correlations was consistent with our VBM analyses above.

Next, we examined correlations between real-world network size score (see §2) and grey matter density at the same set of loci. We found a significant correlation only at the right amygdala (*T*_63_ = 2.17, *R* = 0.264, *p* < 0.05), but not at any other tested loci (left amygdala: *T*_63_ = 1.55, *R* = 0.192, *p* = 0.127; left MTG: *T*_63_ = −0.132, *R* = −0.02, *p* = 0.896; right STS: *T*_63_ = 1.14, *R* = 0.142, *p* = 0.258; right entorhinal cortex, *T*_63_ = 1.20, *R* = 0.150, *p* = 0.234).

Finally, we computed the partial correlation between grey matter at each locus and FBN while regressing out the factor of real-world social network size score (see §2). Even after regressing out the contribution of real-world network size, we found significant correlations at left MTG (*T*_62_ = 2.284, *R* = 0.279, *p* < 0.05), right STS (*T*_62_ = 2.453, *R* = 0.298, *p* < 0.05) and right enthorinal cortex (*T*_62_ = 2.222, *R* = 0.272, *p* < 0.05). However, we did not find significant correlations at amygdala in this sample (left: *T*_62_ = 0.709, *R* = 0.09, *p* = 0.481; right: *T*_62_ = 0.836, *R* = 0.11, *p* = 0.406). We also computed partial correlation between real-world social network size and grey matter density while regressing out the factor of FBN. This also showed significant correlation at right amygdala (*T*_62_ = 2.410, *R* = 0.293, *p* < 0.05), but nowhere else (all *p* > 0.05).

These results together suggest that the three cortical loci, i.e. left MTG, right STS and right entorhinal cortex, are specifically associated with *online* social network size, whereas the right amygdala is associated with *real-world* social network size, while these two variables exhibited some degree of correlation (figure 3).

## 4. Discussion

Taken together, our findings show that the number of social contacts declared publicly on a major web-based social networking site was strongly associated with the structure of focal regions of the human brain. Specifically, we found that variation in the number of friends on Facebook strongly and significantly predicted grey matter volume in left MTG, right STS and right entorhinal cortex. These findings survived whole-brain correction for multiple comparisons and replicated in an independent sample. Moreover, we found that the grey matter density of the amygdala, which was previously shown to be linked with real-world social network size, was also correlated with online social network size. While our correlation analysis precluded definitive ascription of cognitive functions to the areas that predicted the number of online social contacts, it is nevertheless striking that both areas have been implicated in social aspects of human cognition in rather different contexts.

Activations of the regions in the posterior STS are commonly implicated in the perception of biological motion, such as actions executed by others, including hand movement [37,38], direction of gaze [39,40] and mouth movement [39] (see [8] for a review). However, posterior STS is also sensitive to other people's intentions [41–43] as well as navigation in social networks [44], suggesting a possible role of this region in more than just recognizing bodily movements of others. Moreover, differences in cortical anatomy in very similar areas can be seen in disorders where impaired social relationships are recognized [45,46].

On the other hand, we did not find association with online social network size in the fronto-parietal cortical circuit implicated in the mirror mechanism for understanding actions and intentions of other agents [13], nor did we find associations in the medial prefrontal cortex or in TPJ, regions implicated in mentalizing and perspective-taking [12,15,47–51]. Some caution is warranted in interpreting this negative finding, given our relatively conservative statistical criterion reflecting the exploratory nature of the analysis. Nevertheless, taken together with our finding that posterior STS and MTG were correlated only with online social network size, but not with real-world network size (see experiment 4), we suggest that the size of online social networks may reflect specific types of social cognition supported by posterior STS and MTG.

In addition to brain regions implicated in various aspects of social cognition, we found that the right entorhinal cortex was correlated with online social network size. The right entorhinal cortex is implicated in associative memory formation for pairs of items including pairs of names and faces [18,52,53]. Such memory capacity for name–face associates would constitute an important function for maintaining a large social network as observed in social network websites. The lack of correlation with real-world social network size in this area suggests that the involvement of the memory-related region may be specific to the large size of online social networks.

Variability in the size of real-world social networks, as previously observed [33], was also correlated with variability in the size of online networks. This supports the claim that most Internet users employ online social network services to support pre-existing social relationships, maintaining, reinforcing or otherwise solidifying existing offline relationships [2,54]. The cross-scale correlation of social network we observed supports the idea that the FBN, which is probably driven by weak ties, is predictive of the real-world social network size of an individual defined by more intimate friendships. However, the grey matter density at the loci identified by our VBM analysis did not correlate with the real-world social network size, but was specifically associated with the FBN (see experiment 4). On the other hand, the amygdala was associated with the real-world network size more than the online network size, replicating recent work relating real-world social network size to amygdala volume [6]. The differential weights of the neural loci for online and real-world network size suggest that online and real-world network size reflect different aspects of socio-cognitive functions, mediated by posterior STS/MTG and amygdala, respectively.

We deliberately chose to study a population consisting largely of college students, because such individuals almost invariably show a high usage of social-networking sites that are integrated into their existing networks [2,54]. In support of this notion, we found that the size of online friendship networks was correlated with the size of more intimate real-world social networks (figure 3). Nonetheless, it remains to be seen whether such a strong predictive relationship also holds for other age or demographic groups.

A relationship between cortical volume and social network size has been observed across different species of primates [4,5,55]. However, such earlier analyses were limited to global cortical volume and could not specify which brain structures might be associated with large social network sizes in humans. Our VBM results now suggest that the posterior STS and MTG, implicated in social perception, and the entorhinal cortex, implicated in associative memory, provide the cognitive capacity to build and maintain large online social networks in human society. Moreover, they show a link between variability in structural (rather than functional) properties of the human brain and online social network size.

Finally, our study was by design cross-sectional and so cannot determine whether the relationship between brain structure and social network participation arises over time through friendship-dependent plasticity in the brain areas involved; or alternatively whether individuals with a specific brain structure are predisposed to acquire more friends than others. The relative contributions of ‘nature’ and ‘nurture’ therefore remain to be determined.
